# Assessment of the Alveolar Capillary Network in the Postnatal Mouse Lung in 3D Using Serial Block-Face Scanning Electron Microscopy

**DOI:** 10.3389/fphys.2019.01357

**Published:** 2019-11-21

**Authors:** Tobias Buchacker, Christian Mühlfeld, Christoph Wrede, Willi L. Wagner, Richard Beare, Matt McCormick, Roman Grothausmann

**Affiliations:** ^1^Institute of Functional and Applied Anatomy, Medizinische Hochschule Hannover, Hanover, Germany; ^2^Biomedical Research in Endstage and Obstructive Lung Research (BREATH), Member of the German Center for Lung Research, Hanover, Germany; ^3^REBIRTH Cluster of Excellence, Hanover, Germany; ^4^Research Core Unit Electron Microscopy, Hannover Medical School, Hanover, Germany; ^5^Department of Diagnostic and Interventional Radiology (DIR), University of Heidelberg, Heidelberg, Germany; ^6^Translational Lung Research Center (TLRC), Member of the German Center for Lung Research (DZL), University of Heidelberg, Heidelberg, Germany; ^7^Department of Medicine, Monash University, Melbourne, VIC, Australia; ^8^Developmental Imaging, Murdoch Children's Research Institute, Melbourne, VIC, Australia; ^9^Kitware, Inc, New York, NY, United States

**Keywords:** serial block-face scanning electron microscopy, lung, capillary network, 3D reconstruction, segmentation

## Abstract

The alveolar capillary network (ACN) has a large surface area that provides the basis for an optimized gas exchange in the lung. It needs to adapt to morphological changes during early lung development and alveolarization. Structural alterations of the pulmonary vasculature can lead to pathological functional conditions such as in bronchopulmonary dysplasia and various other lung diseases. To understand the development of the ACN and its impact on the pathogenesis of lung diseases, methods are needed that enable comparative analyses of the complex three-dimensional structure of the ACN at different developmental stages and under pathological conditions. In this study a newborn mouse lung was imaged with serial block-face scanning electron microscopy (SBF-SEM) to investigate the ACN and its surrounding structures before the alveolarization process begins. Most parts but not all of the examined ACN contain two layers of capillaries, which were repeatedly connected with each other. A path from an arteriole to a venule was extracted and straightened to allow cross-sectional visualization of the data along the path within a plane. This allows a qualitative characterization of the structures that erythrocytes pass on their way through the ACN. One way to define regions of the ACN supplied by specific arterioles is presented and used for analyses. Pillars, possibly intussusceptive, were found in the vasculature but no specific pattern was observed in regard to parts of the saccular septa. This study provides 3D information with a resolution of about 150 nm on the microscopic structure of a newborn mouse lung and outlines some of the potentials and challenges of SBF-SEM for 3D analyses of the ACN.

## 1. Introduction

The design of the gas-exchange region of the adult mammalian lung combines a large epithelial and endothelial surface area and a thin tissue barrier for optimized gas diffusion (Gehr et al., 1978; Maina and West, 2005). The fetal development of the lung is characterized by the subsequently occurring pseudo-glandular, canalicular, and saccular stages. The saccular phase is the first where sufficient gas exchange is possible and is followed by the alveolar stage during which mature alveoli are formed. Species differences exist with regard to onset of alveolarization before or after birth. In rodents, for example, the alveolar phase starts about 3 days after birth whereas in humans the alveolarization begins around the 36th week of gestation and lasts until postnatal growth has been finished. Parallel to the alveolarization, the maturation of the microvasculature takes place (Burri, 1975; Smith et al., 2010; Schittny, 2017).

During the saccular and early alveolar phase, the alveolar capillary network (ACN) consists of two layers of capillary sheets whereas in the mature lung only one sheet of capillaries forms the ACN in the alveolar septum (Burri, 1975; Caduff et al., 1986). The double-layered ACN in the developing lung has long been regarded as the prerequisite for the formation of new alveoli although lung growth after micro-vascular maturation, i.e., after pneumonectomy, suggests that this may not be a necessity (Hsia et al., 1994; Fehrenbach et al., 2008). Anyway, evidence exists that the vasculature is of particular importance for the development and maintenance of the alveolar septum: Bronchopulmonary dysplasia (BPD) develops as a consequence of preterm birth and interrupted or aberrant lung development (Solaligue et al., 2017). Several authors have hypothesized that the disrupted alveolarization is the result of a disrupted micro-vascular development, the so-called vascular hypothesis of BPD (Klekamp et al., 1999; Bhatt et al., 2001; Maniscalco et al., 2002). Similarly, a vascular hypothesis has been proposed for COPD/emphysema based on the pathological observation that alveolar septa were present in emphysematous lungs which were devoid of capillaries suggesting that loss of capillaries precedes the loss of alveolar septa/epithelium (MacNee, 2005).

The ACN has a complex 3D structure and methods to analyze it comparatively during development and under pathological conditions are needed (Mühlfeld et al., 2018). Ideally, the methods have a high spatial resolution, contain information on the ACN and its micro-environment and are efficient with regards to time and costs. Recently, a formal stereological approach to estimate the number of capillary loops in the ACN was established (Willführ et al., 2015). In addition, a semi-automatic digital tool to generate 3D reconstructions of the ACN from histological sections was developed (Grothausmann et al., 2017). This approach has several limitations, for example the section thickness, sectioning artifacts, and the resolution of the light microscope. Particularly, in the developing lung, the double-layered ACN as well as the proposed mode of angiogenesis (intussusception) (Burri and Djonov, 2002) require a method of higher resolution to image even very thin cellular structures of a few hundred nanometer thickness.

Serial block-face scanning electron microscopy (SBF-SEM) in combination with digital image analysis offers a method to analyze the ACN of newborn lungs. In contrast to serial sectioning, this technique is based on the repetitive scanning of a block face followed by (automatic) removal of an ultra-thin (approximately 80 nm) section (Peddie and Collinson, 2014; Ochs et al., 2016; Schneider et al., 2019). Scans can run automatically over a few days to weeks and are nearly free of sectioning artifacts. Also, the alignment of the sections requires only minimal effort.

Here, we tested the suitability of SBF-SEM and digital image analysis to visualize the capillary network in the newborn mouse lung. Although there are only sacculi at that age, for the sake of convenience we will use the term ACN also for the newborn capillary network. Special aims were to distinguish between single- and double-layered parts of the ACN, to visualize the transition from arterioles into the ACN and to analyze the parts of the ACN that are potentially perfused from a certain entry of an arteriole. According to developmental studies, one mode of enlargement of the ACN is the in-growth of cellular processes within the existing vessels leading to a lateral expansion of the network. This processes is known as intussusception (Burri and Djonov, 2002). Therefore, it was tested whether presumed intussusceptive pillars can be identified.

## 2. Materials and Methods

### 2.1. Animal and Surgery

A neonatal wildtype mouse (C3B6 F1 C57BL/6 WT mice, Charles River Laboratories, Sulzfeld, Germany) weighing 1.3–1.4 g was deeply anesthetized with an intraperitoneal injection of ketamine and xylazine (120 and 16 mg/kg, respectively) and sacrificed by exsanguination. Lungs were perfusion fixed using an adapted protocol by Vasilescu et al. (2012b). In brief, the trachea was cannulated and lungs were inflated with room air at a constant pressure of 20 cm H_2_O, the pulmonary artery was cannulated via a small incision in the right heart ventricle. A preflush solution consisting of 94.5% isotonic Ringer (with 5% dextran), 5% procaine (10%), and 0.5% heparin was administered via the pulmonary artery at 20 cm H_2_O pressure and allowed to drain through a small incision in the left heart atrium. After 5 ml of preflush perfusion and the lung tissue visually cleared from blood, the perfusion solution was changed to fixative solution containing 1.5% glutaraldehyde and 1.5% paraformaldehyde in a 0.15 M HEPES buffer at pH 7.35. Vascular perfusion fixation was performed for 10 min at constant airway and perfusion pressures. After perfusion fixation the trachea was double-tied with a 5-0 surgical silk, removed and submerged in fixation solution overnight at 4°C. All experiments were approved by the Regierungspräsidium Karlsruhe and were conducted in agreement with national and international guidelines. Mice were housed in a pathogen-free animal facility and had free access to chow and water.

### 2.2. Preparation and Fixation

For 3D SBF-SEM analysis the samples were processed as described in Beike et al. (2019) based on Deerinck et al. (2010b), in brief: After fixation (0.15 M HEPES buffer with 1.5% glutaraldehyde and 1.5% paraformaldehyde, pH 7.35) small blocks of the samples (edge length 1–2 mm) were stained en bloc applying a rOTO protocol (rOTO: reduced osmium tetroxide “thiocarbohydrazide” osmium tetroxide) with uranyl acetate and lead aspartate to obtain enough contrast and conductivity of the biological structures for SEM imaging. The samples were dehydrated in an ascending acetone series and embedded in Durcupan™ ACM resin (Sigma-Aldrich, St. Louis, USA). To prepare the polymerized resin blocks for SBF-SEM, the embedded samples were roughly trimmed, mounted with conductive epoxy glue (Chemtronics, CircuitWorks, Kennesaw, USA) on an aluminum specimen pin (Gatan, Pleasanton, CA, USA) and precisely retrimmed with glass knives. This was followed by sputter coating of the samples with a 20 nm gold layer.

### 2.3. EM-Acquisition

Five thousand two hundred and forty-six sections were cut with 80 nm section thickness in a Zeiss Merlin VP Compact SEM (Carl Zeiss Microscopy GmbH, Jena, Germany) equipped with a Gatan 3View2XP system (Gatan Inc., Pleasanton, CA, USA) and the block-face was imaged with a field of view of 525 × 525 μm (15,000 × 15,000 pixel, 35 nm pixel size, 0.5 μs dwell time) with 3.0 kV acceleration voltage in the variable pressure mode at 30 Pa.

### 2.4. Digital Processing

The digital processing used in this paper is defined and documented in source-code, scripts and Makefiles, their specific dependencies are tracked by git. The repository is available at http://www.gitlab.com/romangrothausmann/17-297e_1_s02. With the Makefiles in this repository it is possible to reproduce data, images, and analyses reported in this article. Based on the segmentations from the semi-automated method described below, GNU Makewas used to automate the execution of various programs in accordance to the dependencies defined in the Makefiles. A Supermicro Server (sysGen, Bremen, Germany) was used with 2 Intel Xeon E5-2667 processors yielding a total of 16x2 cores, 512 GB CPU-RAM, equipped with Nvidia TITAN X (12 GB GPU-RAM) graphic cards for GPU-processing and remote visualization through VirtualGL (Commander, 25). GNU/Linux (Debian 9) was used as operating system, making use of various open-source tools, e.g., ITK, VTK, ITKSnap, ParaView, gnuplot, GNU parallel, and many others (Tange, 2011a; Yushkevich et al., 32; ITK development team, 412; Williams et al., 44; ParaView development team, 531; VTK development team, 81).

The image series from the SBF-SEM (35 × 35 × 80 nm, 16-bit, approximately 2 TB) was resampled to an isotropic voxel size of 150 nm (3,500 × 3,500 × 2,797 voxel, 16-bit, approx. 64 GB), which is sufficient to resolve even the thin diffusion barriers and allows to keep the maximum memory foot-print around the size of the given RAM of 512 GB for the most demanding programs used in this study. The contrast was adjusted slice by slice to a given mean and variance to equalize the variations that occurred over the days of acquisition time.

The digital processing characterizes the sample in the following ways:

- regions of supply of the ACN.
- profile of blood vessel diameter as it transitions from artery to capillary to vein.
- diffusion distances in air and tissue.

These characterizations include the following phases:

- semi-automated segmentation in to blood, air, tissue, and lymph.
- generation of vessel center-line between selected points.
- characterization of vessel radius based on the center-line.
- segmentation of regions of supply.

To ease understanding of the processing descriptions, corresponding figures are referenced where appropriate, possibly in a different order than in section 3.

#### 2.4.1. Segmentation

A semi-automated method was used for the segmentation into air, blood, tissue, and lymph segments. An additional label was used to mark regions that were not clearly assignable to one of these not even with the extra context of the 3rd dimension. A morphological 3D watershed segmentation of the gradient magnitude was loaded into a specially extended version of ITKSnap (for detail see https://github.com/pyushkevich/itksnap/pull/1), that allows to efficiently assign the over-segmented watershed labels to each of the desired categories. It was necessary to divide the dataset into octants in order to keep the memory-footprint below the given RAM for generating the 3D watershed segmentation. Apart from the special processing described below, holes are filled in the air and blood segmentation because neither tissue, air or lymph are expected to “float” in blood nor tissue, blood or lymph are expected to “float” in air.

Lower and upper bounds of the segmentations were created as described in Grothausmann et al. (2017). These are used to obtain bounds for the quantifications in Table 1.

**Table 1:** Measurements of relative volumes and relative surfaces.

	***V*_*low*_**	***V***	***V*_*upp*_**	***S*_*low*_**	***S***	***S*_*upp*_**
B	17.4	18.1	21.8	66.7	69.2	91.5
A	54.3	54.6	55.0	40.8	43.9	42.5
L	1.1	1.1	1.3	3.0	3.3	4.5
T	27.1	26.2	21.8	105.7	111.2	132.9

*Relative volume (V(x)/V(s) [%]) and relative surface (S(x)/V(s) [mm^2^/mm^3^]) of blood (B), air (A), tissue (T) and lymph (L) and their lower and upper bounds. The reference volume of the sample V(s) is 0.116 mm^3^*.

#### 2.4.2. Processing the Segmentation of the Air and Blood Space

A 3D distance map is created from the segmentation of the air space (a-dm). This is used in **Figure 7d** to color the air segment according to the shortest distance to tissue in 3D.

Based on the qualitative evaluation of the vasculature contained within the dataset, a point in the artery and the vein are used as markers. A geodesic distance map is constructed from the manually selected artery-point to all points in the blood segmentation using fast marching (A-fm) (Sethian, 1999). The values of the resulting A-fm can be interpreted as the shortest distance through the vasculature to the artery-point. The same is done for the vein-point yielding V-fm. Then A-fm is subtracted from V-fm, which results in a dataset (V-A-fm) where points that are equally far away from the artery-point and the vein-point have a value of zero, values closer to the artery-point are positive, those closer to the vein-point are negative.

A signed Euclidean distance map can be generated for the air segmentation and then related to the blood segmentation (t-dm). The resulting dataset can be used to color-code the shortest local distance from the blood surface to the air as in Figure 3A.

For further interpretation, a path from the artery-point to the vein-point through the vasculature is found according to Mueller (2008), **Figures 7a,b**. Some modifications to the original code were necessary in this case (for further details see: https://github.com/InsightSoftwareConsortium/ITKMinimalPathExtraction/issues/61). The extracted path is not bound to the discretization of the data, it tries to follow the center of the local vessel cross-section while short-cutting on a voxel scale to be as smooth as possible. The path can be guided by way-points, for which two segmented RBCs were chosen. In addition, a binary-image from the maximum-inscribed spheres (ms, **Figures 7a,b**) along the path can be created. As the path is mostly centered in regard to the vessel cross-section, the diameter of the maximum-inscribed spheres can be interpreted as the local minimal diameter of the vessel. If the vessel cross-section is circular, this diameter represents the vessel diameter.

It is possible to straighten each of the above datasets along this path as described in Grothausmann et al., 2017 using a modified version of the code described in Velut (2011). A center slice of the resulting straightened reformatted volume (SRV) then shows the profile of the vasculature and the surroundings along the path. **Figure 7c** shows a center slice of the SRVs of the gray-value dataset with a semi-transparent overlay of the blood, air, and lymph segmentation. The two RBCs used as way-points are centered in the vessel profile. Additionally, the SRV of the ms binary-image is overlaid as well. In regions where this matches the blood segmentation the local circular cross-section is close to circular, in the other regions the difference can be seen as an indicator for non-circular vessel cross-sections.

A slice from the SRVs of V-A-fm, a-dm, and t-dm are shown in **Figure 7d**. Different color look-up-tables (LUTs) were used to indicate the range of distance values according to each dataset. V-A-fm ranges from red (artery-point) to blue (vein-point) over magenta (same distance to artery and vein-point); a-dm ranges from green (proximal) to black (distal); t-dm ranges from yellow (close to air) to black.

**Figure 1:**
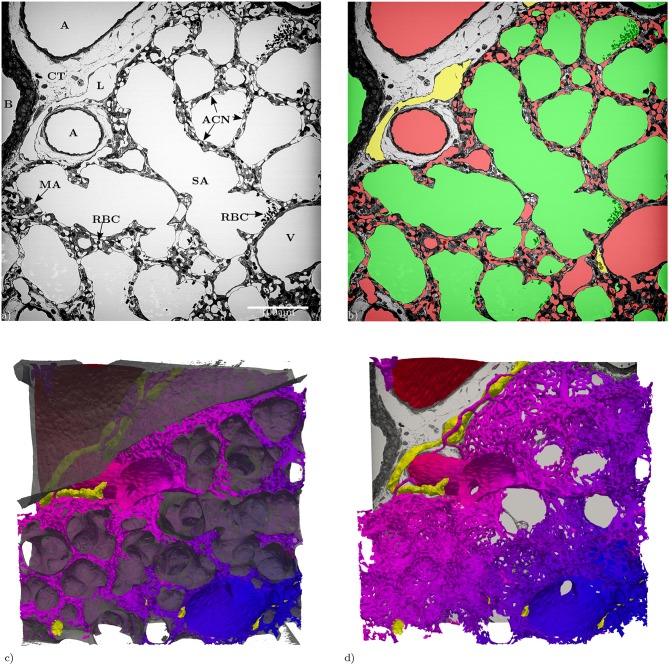
**(a)** Slices of the entire tissue block (525 × 525 μm) in the z-direction: A, artery; ACN, alveolar-capillary network; B, bronchiolus; CT, connective tissue; RBC, red blood cells; L, lymphatic vessel; MA, microatelectasis; SA, saccular airspace; V, vein. **(b)** Segmentation of the structures of interest (same slice as in **a**): red = blood vessels, green = airspace, yellow = lymphatic vessels. **(c)** 3D Surface rendering (525 × 525 × 420 μm) of the segmented structures of interest, air: semi-transparent gray, vasculature: red to blue, lymphatic vessels: yellow. **(d)** Like **(c)** without the air surface but with the slice from **(a)**.

It is possible to separate the larger blood vessels from the ACN with a morphological opening. Regions, a ball with a radius of 8 μm can reach, are regarded as larger vessels, the rest as ACN. The value of 8 μm was chosen as a trade-off between removing non-ACN vessels and preserving wider vessel segments contained within the ACN. Labeling the connected components of the ACN binary image (ACN-lcc) shows that there is one main part (consisting of about 2·10^9^ voxel), all other parts have less than about 4·10^6^ voxel (as e.g., the ACN visible in the upper left corner of Figure 1c).

There seem to be a few hundred entries from the arterioles into the ACN. Even manual identification of these is difficult. Markers for these were therefore created by using the local maximum inscribed sphere radius as an indication to such entries. These markers where then used for a morphological watershed transform in order to investigate the “region-of-supply” (ROS) of the ACN of each of these entries. The 3D morphological watershed transform basically floods the vasculature in parallel starting from each marker. Then, the region flooded by a marker indicates its ROS. **Figure 6A** shows a few of such ROSs which can vary significantly in size and shape. The relationship between cross sectional area of the ACN and distance to the ROS marker can be examined via the histogram of the geodesic distance map from the ROS markers (similar to A-fm). The count of voxels at each distance is an approximation of the cross sectional area, and the likely bounds on the approximation can be evaluated by comparing the minimal projected area of a voxel (the smallest face) to the maximum projected area (along the voxel diagonal, **Figure 6B**).

#### 2.4.3. Processing the Segmentation of the Septal Walls

A segmentation of the septal walls can be created by combining the segmentation of the tissue with the ACN (and the lymph vessels). These septal walls can then be split into two sides by a morphological watershed on the signed distance map of this segmentation. Parts of the borders of these watershed labels lie in the septal walls. These parts are centered in the septa, i.e., they can be regarded as center surfaces (cS) of the septa (Figure 3B)^1^. The cS can be used to split the septa and the ACN. For a double-layered ACN, this should lead to a single-layered ACN for each region (sacculus) (see **Figure 4D**). Furthermore, the intersection of the double-layered ACN with the cS can be regarded as the inter-communication between the two layers of the ACN, **Figure 4A**. For a single-layered ACN, the cS would split each ACN segment into a “half tube.” Whereas, the sides of a double-layered ACN would mostly consist of intact tubes, only the segments connecting both sides would be cut. Therefore, the surface of the cS inside the ACN can be seen as the inter-communication surface, see red regions on the otherwise blue cS in **Figure 4A**. The ratio of the inner surface (red) to the whole cS surface (red and blue) could be a measure for the inter-communication between two adjacent ACN layers. The ratio is about 20% for the analyzed sub-region. However, this measure is expected not to decrease for a single-layered ACN but to increase because the cS then intersects the whole ACN and not just the inter-communication segments. Examining the ACN at the intersection with the cS, **Figures 4B,C**, show, that transitions from single to double-layered ACN exist and that inter-communication segments are not necessarily perpendicular to the cS.

Probing the signed distance map of the septal walls with the cS can be used to visualize the local thickness of the septa (see Figure 3B). A histogram of these values then represents the distribution of the septal wall thickness (Figure 3C). A visualization of the shortest local diffusion distance from air to blood can be created by probing the signed distance map of the septal walls with the ACN (Figure 3A). However, it should be noted that this should be interpreted with caution because its meaning for the inner sides of the double-layered ACN is unclear. Therefore, the distribution of the values is not shown but it can be expected that the distribution would change significantly for a single-layered ACN.

## 3. Results

**Figure 2:**
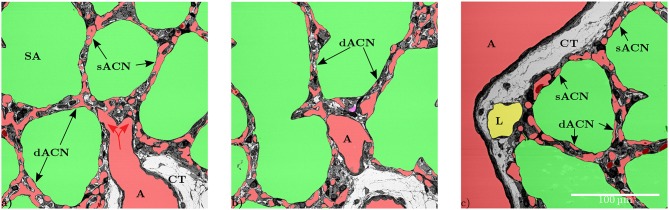
**(a)** The artery (A) is surrounded by a single layer of smooth muscle cells and loose connective tissue (CT). The vessel splits into smaller vessels (red arrows) that are no longer surrounded by connective tissue and form the capillary network in the inter-saccular septa. This artery provides blood for several adjacent sacculi. In this slice some parts of the inter-saccular ACN only contain a single layer of capillary vessels (sACN), while other parts contain a double-layered ACN (dACN). **(b)** Shows the same septa in a different slice, where it can be observed that there also is a double-layered capillary network (dACN) in these septa. The magenta part of the segmentation in the middle of this figure could neither be assigned to the blood vessels, the airspace nor the lymphatic vessel with certainty. **(c)** Slices where all inter-saccular septa contain a double-layered capillary network (dACN), while the septa that are located between the sacculi and non-parenchymal structures, e.g., lymphatic (L) or artery (A), only contain a single layer of capillaries (sACN).

**Figure 3:**
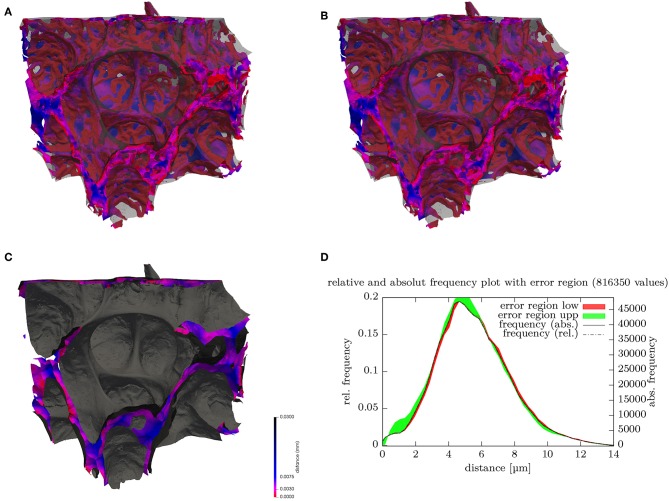
**(A)** Surface rendering of air and blood segmentation contained within a 180 × 152 × 158 μm subregion. The ACN surface is color-coded (key shown in **C**) according to the local minimal distance to the air surface, which is rendered in transparent gray, **(B)** stereo image pair for **(A)**. **(C)** Surface rendering of air (gray) and the center surfaces (cS) inside septal walls color-coded. **(D)** Distribution (histogram) of the septal wall “radius” from **(B)** i.e., shortest distance of cS to air surface. Disregarding possibly differing directions on each side, twice the value can be regarded as the local septal wall thickness. The frequency relates to the number of vertices of the surface mesh. The distributions for the lower (red) and upper (green) bounds segmentations give an indication of the error ranges.

**Figure 4:**
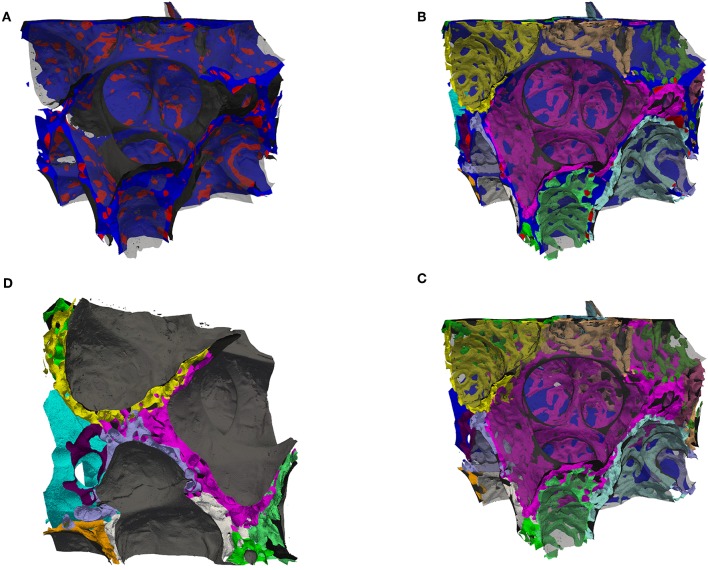
**(A)** Surface rendering of air and the septal center surfaces (cS, as in Figure 3B) here colored such that the surface is blue inside tissue and red inside the ACN, i.e., the red regions are the intersection of the cS with the ACN. The air surface is rendered in transparent gray. **(B)** Surface rendering of air (transparent gray), the center surfaces (cS, as in **A**) and the ACN, which is arbitrarily colored such that each side (in regard to cS) has a different color. **(C)** Same as **(B)** but without the cS, instead the vein in blue (partially contained in the back). This view allows to distinguish the single-layered ACN toward the vein and the double-layered ACN mostly in septa separating sacculi. **(D)** Similar to **(B,C)** but viewed from a side, intransparent gray for the air surface to highlight the splitting of the double-layered ACN.

**Figure 5:**
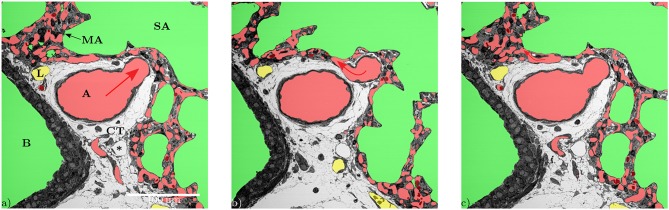
Series of different slices from the z-direction showing the division of an artery (A). While the direction of blood flow (red arrows) can be followed as long as the diameters of the off-branching vessels get smaller **(a,b)**, it becomes unclear within the ACN, especially when capillaries that branch off flow back together **(c)**.

**Figure 6:**
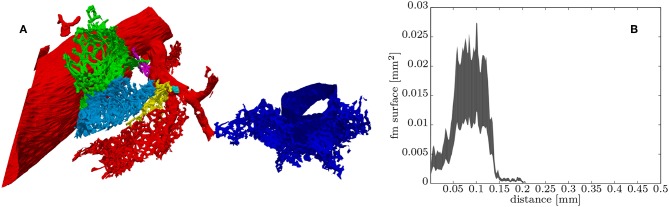
**(A)** Surface rendering of some “regions-of-supply” of the ACN (ROS) each colored differently, the red ROS contains part of the artery and the blue ROS part of the vein. **(B)** Histogram of the distance values contained in the green ROS, which can be roughly related to the upper and lower bounds of the distribution of the total cross-sectional surface within this ROS starting from the entry from the arteriole into the ACN (see section 2.4.2 for details).

**Figure 7:**
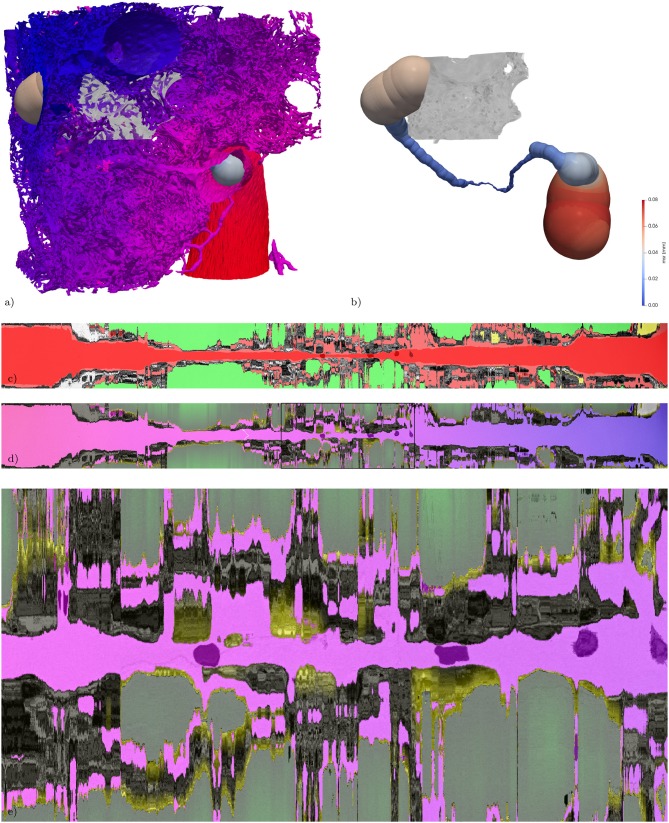
Extracted path from artery to vein and corresponding straightened reformatted volumes (SRVs). **(a)** Surface of the vasculature (as in Figure 1c) combined with a rendering of the maximum-inscribed spheres (ms) along the extracted path. In addition the subregion from Figure 3 is volume rendered in gray-scale. **(b)** Same as **(a)** without the vasculature, revealing the full path indicated by its ms which are colored according to their radius (msr). The path is about 1.2 mm long and the maximum msr along the path is about 76 μm and the minimum msr about 1 μm. **(c)** The SRV of the original data (gray) is overlaid by the segmentation (semi-transparent, air: green; blood: red; lymph: yellow, other: magenta) in addition the voxelization of the locally maximum inscribed spheres is overlaid again in semi-transparent red, giving an indication of local divergence from circular cross-sections. The two RBCs used as way-points for the path extraction are visible as darker spots covered by semi-transparent red along the center line. **(d)** The SRV of the original data (gray) is overlaid by the V-A-fm, a-dm, and t-dm datasets. V-A-fm indicating the distance from the artery to the vein, ranging from red over magenta to blue; a-dm the shortest diffusion distance in the air space towards the surface of the tissue [green (> 50 μm) to black (0 μm)]; t-m the shortest diffusion distance from the surface of the tissue to the blood vessels [yellow (0 μm) to black (> 3 μm)]. **(e)** ACN region marked with a rectangle in **(d)**.

The data set generated by SBF-SEM had a size of 525 × 525 × 420 μm which is approximately 0.116 mm^3^. The majority of the sample consists of the sacculi and their walls including the capillary network. Most of the capillaries are widely open and contain only very few red blood cells (RBCs, erythrocytes). Although most of the sacculi are well inflated, in some cases microatelectases with one or two collapsed sacculi can sometimes be observed and recognized by the thickened septa and multi-layered capillary network. Some sacculi contain extra-vasated RBCs as a preparation artifact, probably originating from perfusion fixation. In addition to the sacculi and their septa, the sample contains both artery and vein. The artery runs adjacent to a bronchus located at the edge of the tissue block and splits into at least 3 arterioles with a diameter of approximately 75 μm that connect with the ACN. The vein has a diameter of approximately 100 μm and has no connection to a bronchus. Figure 1 presents a 3D overview of the vascular structures contained in the tissue block and a representative image from the z stack to demonstrate the fixation quality and content of the sample. Arterioles are surrounded by one layer of smooth muscle cells and loose connective tissue, see Figure 2. The next branching generation of approximately 30–35 μm diameter has only very little surrounding connective tissue and is neighbored by sacculi and their walls. These arterioles branch again into smaller vessels that enter the saccular walls and supply the ACN in various directions. Most parts of the ACN match the concept of a double-layered capillary network Figures 2–4, however, in some parts of the septa the ACN is reduced to a single layer. In the saccular walls bordering the non-parenchymal structures, e.g., larger blood vessels, lymphatic vessels or airways, there is usually only a single-layered ACN (Figures 2, 4). The two capillary sheets (Fung and Sobin, 1969) of regions with a double-layered ACN have multiple connections between them. Relative volume and surface measurements are listed in Table 1. The ratio of blood surface in regard to air surface (*S*(*B*)/*S*(*A*) ≈ 1.6) is larger than 1 (compare e.g. *S*_*Vc*_/*S*_*Va*_ in Table 4 of Grothausmann et al., 2017) as expected in the case of a double-layered ACN. Figure 5 shows another transition of an arteriole into the ACN. The 3D data allows to follow the blood flow from the arteriole into the first capillaries, afterwards the local direction of flow cannot be determined from the structure. The number of possible ways an RBC can take appears nearly unlimited. When investigating the ROS and ACN-lcc datasets, no evidence was found that ROSs are completely separated from each other, overall the connections between neighboring ROSs appear to the be the same as within a ROS, see Figure 6. This means that, there is no definable part of the ACN that is supplied by a single arteriole because the ACN of multiple sacculi are interconnected and fed by several arterioles. It is not possible to predict the flow direction in the ACN (nor within a ROS) because a point in the ACN can be reached from various arterioles. Examining the SRV dataset as shown in Figure 7c, it can be roughly estimated that around 7 sacculi are passed by an RBC following the extracted path.

**Figure 8:**
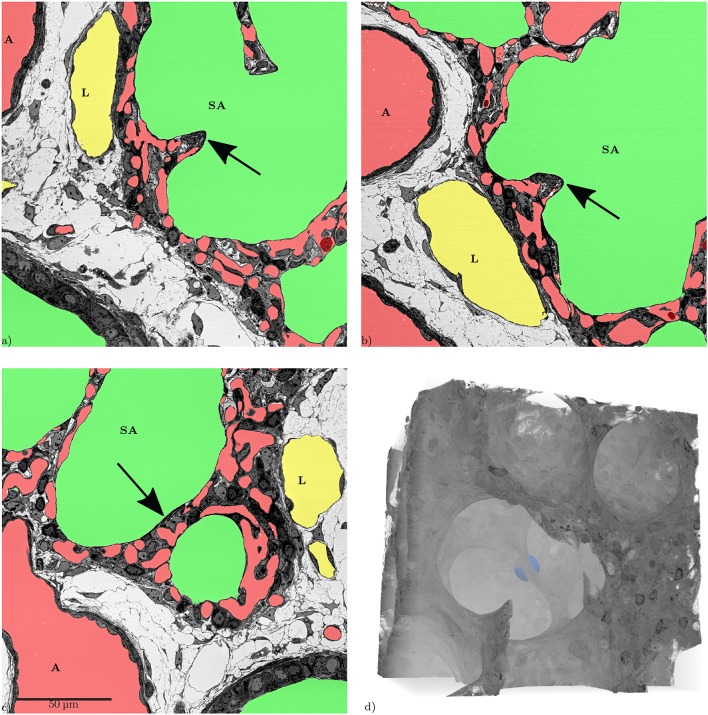
Slices from the xyz-directions, showing the same inter-saccular septum (arrows). The structure in **(a,b)** looks like a “finger-like” upfolding of the septum. **(c)** Shows that this structure is a flat septum that can easily appear to be a so-called “crest” when only looking at it in a single slice. **(d)** 3D volume rendering of the same region, the septum shown in the slices is marked by a blue sphere. The 3D-view reveals that there is no “finger-like” upfolding but a continuous ridge.

**Figure 9:**
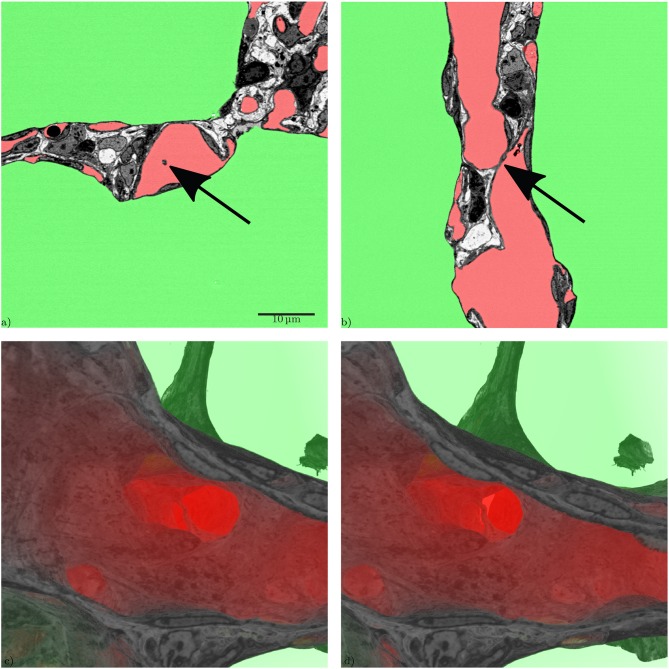
Slices through a tissue pillar, possibly an intussusceptive pillar, from two perspectives, **(a)** z-direction, **(b)** x-direction, **(c)** 3D volume rendering of the subregion containing the structure (tissue: hardly transparent black to gray, blood: very transparent red, air: very transparent green), **(d)** stereo image pair for **(c)**, view behind image plane.

From a developmental point of view, it is desirable to visualize regions of new septal formation and of angiogenesis. The 3D dataset allows analyzing the sample not only in a certain sectional plane but in any selected direction, like in Figure 7. Thus, developing secondary septa, so-called crests or ridges which rise from the primary septa to form new ones, can be looked at in detail, Figure 8. Sprouting and intussusception are potential mechanisms of angiogenesis in the developing lung. Intussusception is thought to occur as a cellular bridge between the two sides of a blood vessel which enlarges by ingrowth of a connective tissue core, leading to tissue pillars in the ACN. Such structures can be found in the dataset, see Figure 9. However, it is difficult to distinguish between an intussusceptive pillar, indicative of the enlargement of the vascular bed, or a regular tissue pillar of the ACN.

## 4. Discussion

By testing the suitability of SBF-SEM for the analysis of the pulmonary capillary network, the present study has provided new 3D information on the structure of the ACN in the newborn mouse lung. In particular, it could be shown that the so-called double-layered capillary network of developing lungs has to be regarded as a single network extending in all three dimensions rather than two (nearly separated) networks within one septum. However, the network contains segments where only a single-layered “two-dimensional” sheet of capillaries is present. As expected, this was regularly observed at the edges of the sacculi adjacent to larger bronchi or arteries but also within regular septal walls. In addition, the study has shown that the ACN of many sacculi is interconnected and receives inflow from various arterioles which adds to the complexity of understanding the blood flow. Because of the enormous surface area of the ACN, only parts of it are required to meet oxygenation demands at rest which has led to the interpretation that only parts of the ACN are perfused at a given point in time (Wearn et al., 1934; Baumgartner et al., 2004; Wagner et al., 2019), the so-called switching between a perfused and non-perfused state. As the mechanism of perfusion switching remains unknown and *in vivo* ACN perfusion is difficult to analyze, the existence of this phenomenon is controversially debated. The diverse sources of arterial inflow in combination with the interconnection among the capillaries of various sacculi (or alveoli) might offer a complex regulatory system at the level of the arterioles. Another functional aspect of this geometry could be safety: If micro-thrombi from the venous system are transported to the lung and settle in the ACN this will only have minimal effects on oxygenation as gas-exchange is only locally diminished as there is no unique downstream part of the network. It also explains why therapeutic approaches using intravenous application of stem cells do not have major adverse pulmonary effects when stem cells get caught in the ACN (Eggenhofer et al., 2012; Lu et al., 2016).

During the analysis of the segmented microvasculature, emphasis was placed on the identification of structural characteristics that have been described in developmental contexts. One such characteristic are intussusceptive pillars. It was originally believed that the alveolarization requires the existence of a double-layered capillary network as the up-folding of one of the two “layers” is the starting point of secondary septa formation (Burri, 1975; Caduff et al., 1986). This double-layered capillary network is believed to be formed by intussusceptive angiogenesis (Schittny et al., 2008; Ackermann et al., 2013). More recent work has led to the concept that even after micro-vascular maturation, alveolarization continues to take place and that a new layer of the ACN is rather formed by sprouting than intussusception (Schittny et al., 2008; Schittny, 2018). Tissue pillars that match the description of intussusceptive pillars can be found in the presented dataset (Figure 9), but their exact role in angiogenesis and lung development is still unclear (Spiegelaere et al., 2012).

Septal up-foldings, so-called ridges that rise from the saccular septa and subdivide the airspace, can easily be identified in the dataset. The reason they often have been described as slender or finger-like protrusions is probably due to their appearance in 2D-analyses (Amy et al., 1975; Zeltner and Burri, 1987), but they appear as long, continuous ridges in 3D that contain a double-layered ACN (Schittny et al., 2008; Branchfield et al., 2016) (Figure 8). It was not possible to observe any characteristic differences regarding the ACN in these ridges nor correlation with the occurrence of intussusceptive pillars. The major aim of the present study was to establish SBF-SEM imaging of relatively large samples of lung parenchyma and to test their suitability for the analysis of the pulmonary microvasculature. Therefore, several aspects of the technique are discussed in the following:

In general, the SBF-SEM requires an embedding procedure that differs from classical embedding techniques for SEM or TEM (Deerinck et al., 2010a; Togo et al., 2014). Before starting the automatic SBF-SEM run for obtaining the data set, semi- and ultra-thin sections were generated from several tissue blocks as quality controls. The ultra-thin sections were examined with a transmission electron microscope to make sure that the rOTO embedding protocol with reduced osmium tetroxide, thiocarbohydrazide and osmium tetroxide had generated sufficient contrast for further SEM analysis (Ochs et al., 2016). The semi-thin sections were used to select a sample with a well-perfused ACN and a larger artery and vein but without a bronchus in the center of the section. Although this procedure helps to find a suitable sample there is no guarantee that the block contains suitable structures in the depth of the sample. Generation of unsuitable data sets due to the unpredictable structural details within the sample is time-consuming and frustrating. Therefore, future studies might benefit from scanning of the sample with a μCT, if available, to make sure the selected block is suited for the intended analysis.

One of the prerequisites of the semi-automatic segmentation used in the present study is the quality of perfusion fixation (Willführ et al., 2015; Mühlfeld et al., 2018). When capillaries are collapsed, the connection of parts of the ACN becomes hardly visible. At the chosen sample size and resolution of the present study, however, even an experienced observer would not be able to identify such lost connections. With the described segmentation approach, RBCs contained in the ACN cause interruptions of the ACN segmentation because the RBCs have a contrast similar to the endothelial cells. In the present study, the combination of automatic pre-segmentation and manual post-segmentation to handle remaining RBCs in the segmentation proved to be a convenient yet still time-consuming method. While the computation of the pre-segmentation lies in the range of a few hours, manual post-segmentation including visual quality control took about two weeks, which is about the same time needed for the SBF-SEM acquisition and for the final digital post-processing. With further advances in machine learning, the recognition and labeling of remaining RBCs could be performed by a trained neural network such that manual interaction could be avoided. Perfusion fixation of neonatal mouse lungs is technically demanding due to the small size of the mouse and its organs. Unfortunately, there is no gold standard for lung fixation (Hsia et al., 2010). Lung fixation is always a compromise and the ideal airway and vascular pressures in a given experimental setting have to be determined for each experimental purpose. Although the chosen airway inflation and vascular perfusion pressures resulted in a very good preservation of the lung tissue with both inflated airways and widely opened capillaries, the occurrence of a few microatelectases and collapsed capillaries could not be prevented completely.

Several methods to visualize and analyze the ACN have been established over the recent decades. Corrosion casts have the advantage that large parts of the vasculature can be viewed, however, as the technique requires the removal of the surrounding tissue, the cellular environment of the blood vessels cannot be visualized (Caduff et al., 1986; Föhst et al., 2015). X-Ray computer tomography (μCT) datasets on the other hand prevent the loss of surrounding structures, but still suffer from low resolution that does often not allow the analysis of cellular details (Vasilescu et al., 2012a; Clark and Badea, 2014), however synchrotron CT can provide higher detail (Xu et al., 2012). The present study has shown that SBF-SEM has the potential of having a firm part in the analysis of the pulmonary microvasculature due to its “open view” (all surrounding structures are visible) and its high resolution. A disadvantage of the SBF-SEM approach is the current limitation of the sample volume which is much smaller than with the other techniques and is destructive. Of course, it would be desirable to investigate a complete, closed circuit within the network and perform flow simulations but such a circuit appears to be too large for the SBF-SEM. Possibly, a combination of subsequently applied different techniques, like μCT and SBF-SEM, may help to overcome the shortcomings associated with each method alone.

In summary, the present study has shown that SBF-SEM is a suitable method to analyze the 3D architecture of the pulmonary micro-circulation, particularly the ACN. Digital segmentation helps to visualize and analyze large volumes of the ACN. The high resolution of the images allows viewing small-sized structures like endothelial bridges indicative of intussusceptive angiogenesis. It may be of particular usefulness in the comparative analysis of normal and pathologically altered capillary networks.

## Data Availability Statement

The git repository containing Makefiles for automated reproduction of the image processing used for this manuscript is available at http://www.gitlab.com/romangrothausmann/17-297e_1_s02. All used software is open-source and freely available, programs and modifications specifically created for the presented analyses can be found at http://www.github.com/romangrothausmann/ and http://github.com/richardbeare/, docker images of these in the corresponding registry at http://www.gitlab.com/romangrothausmann/. The datasets generated for this study are available on request to the corresponding author.

## Ethics Statement

The animal study was reviewed and approved by Regierungspräsidium Karlsruhe.

## Author Contributions

TB, CM, and RG conceived and designed the research, conducted the study, drafted the manuscript, and prepared the figures. WW performed the sample preparation. CW performed the EM-acquisition. RG, RB, and MM performed digital processing and necessary programming. All authors have edited, critically revised, and approved the final version of the manuscript.

### Conflict of Interest

MM was employed by Kitware, Inc. The remaining authors declare that the research was conducted in the absence of any commercial or financial relationships that could be construed as a potential conflict of interest.

## Footnotes

^1^This approach for the creation of the cS does not create center surfaces for septa that do not lead to a 3D constriction of saccular entrances, as can be seen in Figure 4 for the inner septa in the image center. Another way to construct a cS is to use 2D skeletons processed slice by slice. This method does not suffer from the mentioned limitation, but even if done for each spatial direction and combined, it leads to far more inappropriate extrusions than the watershed approach and the cS is likely to have holes.

## References

[B1] Ackermann M. Houdek J. P. Gibney B. C. Ysasi A. Wagner W. Belle J.. (2013). Sprouting and intussusceptive angiogenesis in postpneumonectomy lung growth: mechanisms of alveolar neovascularization. Angiogenesis 17, 541–551. 10.1007/s10456-013-9399-9 24150281 PMC4061467

[B2] Amy R. Thurlbeck W. Bowes D. (1975). Post-natal growth of the mouse lung. Tubercle 56:241 10.1016/0041-3879(75)90060-4

[B3] Ayachit U. (2016). The ParaView Guide, Community Edn. Kitware Inc.

[B4] Baumgartner W. A. Peterson A. J. Presson R. G. Tanabe N. Jaryszak E. M. Wagner W. W. (2004). Blood flow switching among pulmonary capillaries is decreased during high hematocrit. J. Appl. Physiol. 97, 522–526. 10.1152/japplphysiol.00068.2003 15247197

[B5] Beike L. Wrede C. Hegermann J. Lopez-Rodriguez E. Kloth C. Gauldie J.. (2019). Surfactant dysfunction and alveolar collapse are linked with fibrotic septal wall remodeling in the TGF-β1-induced mouse model of pulmonary fibrosis. Lab. Invest. 99, 830–852. 10.1038/s41374-019-0189-x 30700849

[B6] Bhatt A. Pryhuber G. Huyck H. Watkins R. Metlay L. Maniscalco W. (2001). Disrupted pulmonary vasculature and decreased vascular endothelial growth factor, Flt-1, and TIE-2 in human infants dying with bronchopulmonary dysplasia. Am. J. Respir. Crit. Care Med. 164, 1971–1980. 10.1164/ajrccm.164.10.2101140 11734454

[B7] Branchfield K. Li R. Lungova V. Verheyden J. M. McCulley D. Sun X. (2016). A three-dimensional study of alveologenesis in mouse lung. Dev. Biol. 409, 429–441. 10.1016/j.ydbio.2015.11.017 26632490 PMC4843524

[B8] Burri P. H. (1975). Postnatal growth and maturation of the lung. Chest 67, 2S–3S. 10.1378/chest.67.2_Supplement.2S 1112100

[B9] Burri P. H. Djonov V. (2002). Intussusceptive angiogenesis–the alternative to capillary sprouting. Mol. Aspects Med. 23, 1–27. 10.1016/S0098-2997(02)00096-1 12537983

[B10] Caduff J. H. Fischer L. C. Burri P. H. (1986). Scanning electron microscope study of the developing microvasculature in the postnatal rat lung. Anat. Rec. 216, 154–164. 10.1002/ar.1092160207 3777448

[B11] Clark D. Badea C. (2014). Micro-CT of rodents: state-of-the-art and future perspectives. Phys. Med. 30, 619–634. 10.1016/j.ejmp.2014.05.011 24974176 PMC4138257

[B12] Commander D. R. (2.5). VirtualGL. Open source.

[B13] Deerinck T. Bushong E. Lev-Ram V. Shu X. Tsien R. Ellisman M. (2010a). Enhancing serial block-face scanning electron microscopy to enable high resolution 3-D nanohistology of cells and tissues. Microsc. Microanal. 16, 1138–1139. 10.1017/S1431927610055170

[B14] Deerinck T. J. Bushong E. A. Thor A. Ellisman M. H. (2010b). NCMIR Methods for 3D EM: A New Protocol for Preparation of Biological Specimens for Serial Blockface Scanning Electron Microscopy. Online (accessed July 31, 2019).

[B15] Eggenhofer E. Benseler V. Kroemer A. Popp F. C. Geissler E. K. Schlitt H. J. (2012). Mesenchymal stem cells are short-lived and do not migrate beyond the lungs after intravenous infusion. Front. Immunol. 3:297 10.3389/fimmu.2012.00297 23056000 PMC3458305

[B16] Fehrenbach H. Voswinckel R. Michl V. Mehling T. Fehrenbach A. Seeger W.. (2008). Neoalveolarisation contributes to compensatory lung growth following pneumonectomy in mice. Eur. Respir. J. 31, 515–522. 10.1183/09031936.00109407 18032439

[B17] Föhst S. Wagner W. Ackermann M. Redenbach C. Schladitz K. Wirjadi O.. (2015). Three-dimensional image analytical detection of intussusceptive pillars in murine lung. J. Microsc. 260, 326–337. 10.1111/jmi.12300 26280540

[B18] Fung Y. C. Sobin S. S. (1969). Theory of sheet flow in lung alveoli. J. Appl. Physiol. 26, 472–488. 10.1152/jappl.1969.26.4.472 5775333

[B19] Gehr P. Bachofen M. Weibel E. R. (1978). The normal human lung: ultrastructure and morphometric estimation of diffusion capacity. Respir. Physiol. 32, 121–140. 10.1016/0034-5687(78)90104-4 644146

[B20] Grothausmann R. Knudsen L. Ochs M. Mühlfeld C. (2017). Digital 3D reconstructions using histological serial sections of lung tissue including the alveolar capillary network. Am. J. Physiol. 312, L243–L257. 10.1152/ajplung.00326.2016 27913424

[B21] Hsia C. C. Herazo L. F. Fryder-Doffey F. Weibel E. R. (1994). Compensatory lung growth occurs in adult dogs after right pneumonectomy. J. Clin. Invest. 94, 405–412. 10.1172/JCI117337 8040282 PMC296323

[B22] Hsia C. C. W. Hyde D. M. Ochs M. Weibel E. R. (2010). An official research policy statement of the American Thoracic Society/European Respiratory Society: standards for quantitative assessment of lung structure. Am. J. Respir. Crit. Care Med. 181, 394–418. 10.1164/rccm.200809-1522ST 20130146 PMC5455840

[B23] ITK development team (4.12). ITK. Open source (see 2456).

[B24] Johnson H. J. McCormick M. M. Ibáñez L. the Insight Software Consortium (2018). The ITK Software Guide. Kitware Inc.

[B25] Klekamp J. G. Jarzecka K. Perkett E. A. (1999). Exposure to hyperoxia decreases the expression of vascular endothelial growth factor and its receptors in adult rat lungs. Am. J. Pathol. Lung Cell. Mol. Physiol. 154, 823–831. 10.1016/S0002-9440(10)65329-1 10079260 PMC1866417

[B26] Lu H. Cook T. Poirier C. Merfeld-Clauss S. Petrache I. March K. L.. (2016). Pulmonary retention of adipose stromal cells following intravenous delivery is markedly altered in the presence of ARDS. Cell Transpl. 25, 1635–1643. 10.3727/096368915X690189 26609693

[B27] MacNee W. (2005). Pathogenesis of chronic obstructive pulmonary disease. Proc. Am. Thorac. Soc. 2, 258–266. 10.1513/pats.200504-045SR 16267346 PMC2713323

[B28] Maina J. N. West J. B. (2005). Thin and strong! The bioengineering dilemma in the structural and functional design of the blood-gas barrier. Physiol. Rev. 85, 811–844. 10.1152/physrev.00022.2004 15987796

[B29] Maniscalco W. M. Watkins R. H. Pryhuber G. S. Bhatt A. Shea C. Huyck H. (2002). Angiogenic factors and alveolar vasculature: development and alterations by injury in very premature baboons. Am. J. Physiol. Lung Cell. Mol. Physiol. 282, L811–L823. 10.1152/ajplung.00325.2001 11880308

[B30] Mueller D. (2008). Fast marching minimal path extraction in ITK. *Insight J*. 1–8. Available online at: http://www.insight-journal.org/browse/publication/213

[B31] Mühlfeld C. Wrede C. Knudsen L. Buchacker T. Ochs M. Grothausmann R. (2018). Recent developments in 3D reconstruction and stereology to study the pulmonary vasculature. Am. J. Physiol. Lung Cell. Mol. Physiol. 315, L173–L183. 10.1152/ajplung.00541.2017 29644892

[B32] Ochs M. Knudsen L. Hegermann J. Wrede C. Grothausmann R. Mühlfeld C. (2016). Using electron microscopes to look into the lung. Histochem. Cell Biol. 146, 695–707. 10.1007/s00418-016-1502-z 27688057

[B33] ParaView development team (5.3.1). ParaView. Open source (see 3).

[B34] Peddie C. J. Collinson L. M. (2014). Exploring the third dimension: volume electron microscopy comes of age. Micron 61, 9–19. 10.1016/j.micron.2014.01.009 24792442

[B35] Schittny J. C. (2017). Development of the lung. Cell Tissue Res. 367, 427–444. 10.1007/s00441-016-2545-0 28144783 PMC5320013

[B36] Schittny J. C. (2018). How high resolution 3-dimensional imaging changes our understanding of postnatal lung development. Histochem. Cell Biol. 150, 677–691. 10.1007/s00418-018-1749-7 30390117 PMC6267404

[B37] Schittny J. C. Mund S. I. Stampanoni M. (2008). Evidence and structural mechanism for late lung alveolarization. Am. J. Physiol. Lung Cell. Mol. Physiol. 294, L246–L254. 10.1152/ajplung.00296.2007 18032698

[B38] Schneider J. P. Wrede C. Hegermann J. Weibel E. R. Mühlfeld C. Ochs M. (2019). On the topological complexity of human alveolar epithelial type 1 cells. Am. J. Respir. Crit. Care Med. 199, 1153–1156. 10.1164/rccm.201810-1866LE 30758981

[B39] Schroeder W. Martin K. Lorensen B. (2006). The Visualization Toolkit: An Object-Oriented Approach to 3D Graphics, 4th Edn. Clifton Park, NY: Kitware Inc.

[B40] Sethian J. (1999). Level Set Methods and Fast Marching Methods: Evolving Interfaces in Computational Geometry, Fluid Mechanics, Computer Vision, and Materials Science. Cambridge Monographs on Applied and Computational Mathematics. Cambridge: Cambridge University Press.

[B41] Smith L. J. McKay K. O. van Asperen P. P. Selvadurai H. Fitzgerald D. A. (2010). Normal development of the lung and premature birth. Paediatr. Respir. Rev. 11, 135–142. 10.1016/j.prrv.2009.12.006 20692626

[B42] Solaligue D. E. S. Rodríguez-Castillo J. A. Ahlbrecht K. Morty R. E. (2017). Recent advances in our understanding of the mechanisms of late lung development and bronchopulmonary dysplasia. Am. J. Physiol. Lung Cell. Mol. Physiol. 313, L1101–L1153. 10.1152/ajplung.00343.2017 28971976

[B43] Spiegelaere W. D. Casteleyn C. den Broeck W. V. Plendl J. Bahramsoltani M. Simoens P.. (2012). Intussusceptive angiogenesis: a biologically relevant Form of angiogenesis. J. Vasc. Res. 49, 390–404. 10.1159/000338278 22739226

[B44] Tange O. (2011a). GNU Parallel - The Command-Line Power Tool. Open source (see 45).

[B45] Tange O. (2011b). GNU Parallel - The command-line power tool. login: The USENIX Magazine 36, 42–47. Available online at: http://www.gnu.org/s/parallel

[B46] Togo A. Ohta K. Higashi R. ichiro Nakamura K. (2014). En blocstaining with hydroquinone treatment for block face imaging. Microscopy 63(Suppl. 1), i34.2–i35. 10.1093/jmicro/dfu078 25359840

[B47] Vasilescu D. M. Gao Z. Saha P. K. Yin L. Wang G. Haefeli-Bleuer B.. (2012a). Assessment of morphometry of pulmonary acini in mouse lungs by nondestructive imaging using multiscale microcomputed tomography. Proc. Natl. Acad. Sci. U.S.A. 109, 17105–17110. 10.1073/pnas.1215112109 23027935 PMC3479519

[B48] Vasilescu D. M. Knudsen L. Ochs M. Weibel E. R. Hoffman E. A. (2012b). Optimized murine lung preparation for detailed structural evaluation via micro-computed tomography. J. Appl. Physiol. 112, 159–166. 10.1152/japplphysiol.00550.2011 21817110 PMC3290416

[B49] Velut J. (2011). A spline-driven image slicer. VTK J. 838:9 Available online at: http://www.vtkjournal.org/browse/publication/838

[B50] VTK development team (8.1). VTK. open source (see 39).

[B51] Wagner W. W. Jaryszak E. M. Peterson A. J. Doerschuk C. M. Bohlen H. G. King J. A. C.. (2019). A perpetual switching system in pulmonary capillaries. J. Appl. Physiol. 126, 494–501. 10.1152/japplphysiol.00507.2018 30571293 PMC6397411

[B52] Wearn J. T. Ernstene A. C. Bromer A. W. Barr J. S. German W. J. Zschiesche L. J. (1934). The normal behavior of the pulmonary blood vessels with observations on the intermittence of the flow of blood in the arterioles and capillaries. Am. J. Physiol. 109, 236–256. 10.1152/ajplegacy.1934.109.2.236

[B53] Willführ A. Brandenberger C. Piatkowski T. Grothausmann R. Nyengaard J. R. Ochs M.. (2015). Estimation of the number of alveolar capillaries by the Euler number (Euler-Poincaré characteristic). Am. J. Physiol. Lung Cell. Mol. Physiol. 309, L1286–L1293. 10.1152/ajplung.00410.2014 26432874

[B54] Williams T. Kelley C. (4.4). gnuplot. open source..

[B55] Xu F. Helfen L. Suhonen H. Elgrabli D. Bayat S. Reischig P.. (2012). Correlative nanoscale 3D imaging of structure and composition in extended objects. PLoS ONE 7:e50124. 10.1371/journal.pone.0050124 23185554 PMC3501479

[B56] Yoo T. Angelini E. Avants B. Aylward S. Chen T. Duda J. (2004). Insight Into Images: Principles and Practice for Segmentation, Registration, and Image Analysis. Weliesey, MA: A K Peters, Ltd.

[B57] Yushkevich P. (3.2). ITK-SNAP. open source (see 58).

[B58] Yushkevich P. A. Piven J. Hazlett H. C. Smith R. G. Ho S. Gee J. C.. (2006). User-guided 3D active contour segmentation of anatomical structures: significantly improved efficiency and reliability. NeuroImage 31, 1116–1128. 10.1016/j.neuroimage.2006.01.015 16545965

[B59] Zeltner T. B. Burri P. H. (1987). The postnatal development and growth of the human lung. II. Morphology. Respir. Physiol. 67, 269–282. 10.1016/0034-5687(87)90058-2 3575906

